# The Opsonic Index of the Blood and the Treatment of Phthisis

**Published:** 1905-11-11

**Authors:** 


### THE OPSONIC INDEX OF THE BLOOD AND THE TREATMENT OF PHTHISIS.

Drs. Lawson and Stewart, Nordrach-on-Dee Sanatorium, contributed an interesting paper to the Medico-Chirurgical Society of Edinburgh on November 1. As the result of the examination of a number of healthy persons, the opsonic index of the blood was found to vary from 9 to 1.2, while in persons suffering from tuberculosis in various forms it was much lower, usually .4 or .5. While not absolutely conclusive, the presence of a high opsonic index was regarded as affording a strong presumption as to the absence of pulmonary affection. This presumption was strengthened if, on the injection of $\frac{1}{500}$ milligram of tuberculin, TR, an immediate rise took place, as in tuberculous patients a fall was observed to follow the injection. The fall of the opsonic index after the injection of tuberculin, termed by Erlich the negative phase, is of very variable duration, and is indicated neither by the pulse, temperature, nor by the subjective sensations of the patient. A fresh injection during this period produced a still further fall in the opsonic index and an increased susceptibility to the disease. After the conclusion of the negative phase there was a gradual rise in the opsonic index, which was increased on repeated injections, so that in a given individual it could be raised to a permanently higher level. In cases where, after prolonged sanatorium treatment, the disease was in a favourable state, the injection of small doses of tuberculin, controlled so as to avoid the negative phrase, raised the opsonic index of the blood to within the limits of health. Drs. Lawson and Stewart maintain that, properly controlled in this way, tuberculin is a valuable aid in the treatment of tuberculosis, but when injected during the negative phase it may do incalculable harm. It is to the absence of this control that they attribute the varying results obtained from the use of tuberculin and the obloquy into which it has fallen.

